# Recreation in Relation to the Health of the People

**Published:** 1891-07-04

**Authors:** 


### Recreation in Relation to the Health of the People.

### V.—RECREATION FOR THE BODY.

No scheme for providing recreation for the working population—at any rate, in London and other large towns—can possibly be considered adequate unless it provides, and provides in large measure, for the recreation of the body as well as of the mind. A large proportion of these people are engaged in more or less sedentary work, which often obliges them to spend most of their time in some constrained position; the rooms in which they work are very generally hot and ill-ventilated; and when they are engaged in more active occupations, it is but seldom that work is to be found which gives an all-round development of the muscles. Besides which, the young, at any rate, are gifted with a superabundant physical energy which even a long and fairly active working-day does by no means exhaust. It has often been observed that the youthful factory girl is always ready for an evening romp in the streets, in spite of having been on her feet all day; and it is just the same with the lads, or to put it into factory-girl language, with the "blokes." It has been well said that during the years between thirteen and seventeen "the physical energies of the body, as in a spring-tide, thrill out into every limb and organ." Some outlet these physical energies must have; and those who recognize this fact, and provide for it by giving facilities to these young people for gymnastics, dancing, swimming, and other physical exercises, do much not only to improve their physical health, but also to guard them against the dangers which would otherwise beset them.

We have abundant testimony as to the former of these two points, both from those concerned in the management of these gymnasia, and from those who are students there—to say nothing of ocular demonstration. To give one instance: The girls attending a musical drill class opened last winter under the auspices of a branch of the Young Women's Christian Association, begged with one accord that it might be re-opened this year, on the very ground that they had felt so much better in health since attending it. "I used to suffer awful with indigestion," said one young dressmaker, "but I hardly ever have it now." "And I," says another, "feel a different creature to what I did before these classes began."

As to the second point—the substitution of healthy for unhealthy amusement, we have been told by East-end workers of considerable experience that they look to gymnastic exercises as a valuable countercheck upon the precocity of the London lad, and the foolish philandering in which he is apt to indulge, for want of something better to do. Is any evidence of this precocity wanted? Here is a brief conversation held, not long ago, in an East-end parish.

*Sunday-school Teacher*: "Johnny, what has become of the other boys in the class? They seem to have given up coming."

*Johnny* (*aged twelve*): "Well, teacher, they mostly go courtin' on Sunday afternoons, see? A year or two ago, I used to go courtin', too, but I've giv' all that up now, and I've took to comin' to school instead."

Truly, if Sunday-school can slay its thousands—we mean its one, it does not seem very unreasonable to hope that a class for gymnastics may slay its tens of thousands.

It is satisfactory to see that the need for gymnastics, both for girls and boys, has been very generally recognised by those who aim at providing them with wholesome recreation. The Recreative Evening Schools Association reports that in nearly every school in which classes have been formed, physical exercise, in some form, is an essential part of the training, and that even postmen and telegraph boys may be seen running and using the dumb-bells with quite fresh vigour. At the People's Palace, again, at Oxford House, Toynbee Hall, the Morley College, the Polytechnic, and last, not least, at Working Lads' and at Factory Girls' Institutes, physical education, in a more or less complete form, is provided for. Thus all classes (for it must never be forgotten that there are various grades in the working population, as among ourselves, and that it is useless to think of catering for it *in the lump*) would seem to have some provision made for them, inadequate though it be. It remains for us to extend that provision; and this, we may remark in passing, may be done, not only by subscriptions, but by personal help. We rather wonder that appeals have to be so constantly made for voluntary helpers in gymnasia for men and boys. It may seem hard upon a man who works with his brains all day to be called upon to spend an hour of his short leisure in teaching geography or book-keeping; but to help in a gymnasium one or two nights in the week would be a thorough change of employment, and, indeed, might prove beneficial to himself as well as to the pupils.

No one can watch the girl-students at the People's Palace at their gymnastic exercises without pleasure and sympathy. Now and again a grand performance or "display" is given in the Queen's Hall, in which assembles a huge and eager throng of the girls' friends and relations—of the gentler sex, the stern announcement being placarded up, "Only Ladies Admitted." Nevertheless, there is generally a sprinkling of shrinking male forms at the platform end of the room; and, terrible to relate, WE have been there!

Most of the students are young sempstresses, dressmakers, and so forth—a class of girls who are apt to be dyspeptic, narrow-chested, and round-backed; but the excellent deportment of these young gymnasts was especially observable. And whether it was the result of pleasurable excitement, of regular gymnastic training, or of the absence of heavy, clinging garments, certain it is that while waiting for the performance to begin, they ran and skipped and capered like playful young lambs. Their first performance, gymnastics on parallel bars, gave one the impression that such exercises were perhaps a little too severe a strain on the delicate physical frame of young girls; but nothing but praise can be given to the rest of the programme.

A prettier sight can hardly be imagined than the long lines of girls, in their simple, dark-blue costumes, rising on their heels and swaying to and fro in rythmical movement, with the long bar-bells now inclining this way, now that, guided by scores of pairs of slender wrists; and when these are exchanged for light dumb-bells, the ear also is charmed by the songs with which the young performers accompany some of the slower and more measured exercises. "Ding-dong Bell" (from "Les Cloches de Corneville") rings out merrily through the room; other exercises go to the well-known airs of "The Boys of the Old Brigade," and "The Union Jack of Old England," and perhaps the greatest favourite of all with the girls (to judge by the way in which they sing it) is "They all Love Jack." We have never been able to understand why whistling should be considered an essentially masculine accomplishment; certainly, it sounds in our ears both feminine and sweet, as the girls tootle the tuneful air of the latter song, only breaking off to inform us, in the words of the chorus, that Jack's "heart is like the sea, ever open, brave, and free."

Swimming must also come in for a word of notice, as being one of the very best forms of physical recreation. It is satisfactory to know that the excellent swimming baths now provided in various parts of London are largely used; and that increasing numbers of school children, to say nothing of adults, now look forward to a weekly swim. An incidental advantage of this is that improved cleanliness is secured, not so much by the actual bath (this might be rather hard upon the body corporate, however beneficial to the individual concerned), as by the shame which the children quickly learn to feel if they come for their lesson in an obviously unwashen condition.

